# Effects of varying dietary folic acid during weaning stress of piglets

**DOI:** 10.1016/j.aninu.2020.12.002

**Published:** 2020-12-23

**Authors:** Lei Wang, Xian Tan, Huiru Wang, Qiye Wang, Pengfei Huang, Yali Li, Jianzhong Li, Jing Huang, Huansheng Yang, Yulong Yin

**Affiliations:** a Laboratory of Animal Nutrition and Human Health, Hunan International Joint Laboratory of Animal Intestinal Ecology and Health, College of Life Science, Hunan Normal University, Changsha, 410081, China; b Hunan Provincial Key Laboratory of Animal Nutritional Physiology and Metabolic Process, National Engineering Laboratory for Pollution Control and Waste Utilization in Livestock and Poultry Production, Key Laboratory of Agro-ecological Processes in Subtropical Region, Scientific Observing and Experimental Station of Animal Nutrition and Feed Science in South-Central, Ministry of Agriculture, Institute of Subtropical Agriculture, Chinese Academy of Sciences, Changsha, 410125, China; c Department of Neuroscience, Hengyang School of Medicine, University of South China, Hengyang, 421000, China

**Keywords:** Folic acid, Growth performance, Antibiotic-free, Intestinal morphology, Post-weaning piglet

## Abstract

The present study was conducted to evaluate the effect of dietary folic acid on the growth performance, intestinal morphology, and intestinal epithelial cells renewal in post-weaning piglets. Twenty-eight piglets (weaned at day 21, initial body weight of 6.73 ± 0.62 kg) were randomly allotted to 4 treatments with 7 pens per diet and 1 piglet per pen. The piglets were fed the same antibiotic-free and zinc oxide-free basal diets supplemented with folic acid at 0, 3, 9, and 18 mg/kg for 14 days. The results showed that dietary supplementation with folic acid increased villus height (VH) (*P* = 0.003; linear, *P* = 0.001), VH-to-crypt depth (VH:CD) ratio (*P* = 0.002; linear, *P* = 0.001), villus surface area (VSA) (*P* = 0.026; linear, *P* = 0.010). The analyzed parameters ADG, serum urea nitrogen (BUN) content, VH, VSA, and serum folate (SF) concentration responded linearly to the dietary folic acid concentration when the dietary folic acid concentration was below 4.42, 5.26, 4.79, 3.47, and 3.53 mg/kg respectively (*R*^*2*^ = 0.995, 0.995, 0.999, 0.999, 0.872, *P* = 0.09, 0.07, 0.09, 0.09, 0.36, respectively), as assessed by a two-linear broken-line regression. Above these breakpoints, the response of ADG, VH, VSA, and SF plateaued in response to changes in dietary folic acid concentration. Moreover, dietary supplementation with folic acid significantly increased the lactase (*P* = 0.001; linear, *P* = 0.001) and sucrase activities (*P* = 0.021; linear, *P* = 0.010) in the jejunal mucosa of weaned piglets. The mRNA expression of solute carrier family 6 member 19 (*SLC6a19*), solute carrier family 1 member 1 (*SLC7a1*), tumor necrosis factor-α (*TNF-α*), the number of Ki67 positive cells, and cell shedding rate had a significant linear contrast (*P* = 0.023, 0.021, 0.038, 0.049, and 0.008, respectively) in dietary folic acid groups. In conclusion, our results indicate that folic acid supplementation can improve the growth performance and intestinal morphology of weaned piglets by maintaining the balance of epithelial cell renewal.

## 1 Introduction

Weaning of piglets is a stressful process associated with gastrointestinal function disorders, and often accompanied by severe growth retardation and diarrhea. It is well established that this process is multi-factorial, and that post-weaning anorexia and under-nutrition are major aetiological factors (Pluske et al., 1995). In order to ensure the healthy growth of weaning piglets, the antibiotics and zinc oxide at pharmacological levels have been widely used as feed additives to improve growth performance of piglets in their transition to the nursery period. However, the abuse of antibiotics increases the antibiotic resistance of bacteria, thereby threatening human health and raising environmental concerns. According to a series of studies, folic acid deficiency can lead to symptoms such as anemia, diarrhea and can reduce weight gain and litter size (Davidson and Townley, 1977; Ashraf et al., 2008). Although the supplementation of folic acid can alleviate chronic diarrhea, folic acid was thought to have no effect on severe cases in children and piglets (Carruthers, 1946; Ashraf et al., 1998).

Folic acid refers to the essential water-soluble vitamin B_9_. Mammals cannot synthesize folic acid, which is indispensable for one-carbon transfer reactions, including nucleic acid synthesis, amino acid metabolism, and methylation (Hayashi et al., 2007). It, therefore, plays a crucial role in protein deposition and tissue synthesis. Folic acid prevents the occurrence of cardiovascular disease, anemia, and fetal development (Matte et al., 1992). Rapidly growing tissues or cells with a high turnover rate are sensitive to folic acid levels; therefore, supplemental folic acid promotes the growth of young animals.

From 1998 to 2012, the National Research Council (NRC) recommended dietary supplementation of folic acid at 0.3 mg/kg of diet for post-weaning piglets (NRC, 1998; NRC, 2012), and the best estimate of the minimum amount required per day for a piglet from 7 to 11 kg BW is 0.14 mg. However, this means the ADFI of piglets is at least 467 g per day, which seems difficult for post-weaning piglets. Folic acid deficiency is caused due to the instability of folic acid and insufficient daily feed intake. Therefore, often, 6 to 10 times of the normal dosage is administered to ensure a safe margin. There are substantial studies on the effects of maternal supplementation of folic acid on reproductive performance and offspring (Rerat, 1978; Matte et al., 1984; Lindemann and Kornegay, 1989; Joshi et al., 2004; Liu et al., 2011a). Yu et al. (2010) showed that dietary supplementation with a high level of folic acid (2.5 mg/kg) improved the growth performance of weaned piglets that were fed a diet with 0.30% zinc oxide. The intestines of post-weaning piglets grow faster compared to their other body parts. Therefore, the proliferation rate of intestinal cells, which is more sensitive to folic acid concentration, is higher than that of other body parts. Moreover, weaning stress has negative effects on the intestinal morphology and functions, resulting in villus atrophy, crypt hyperplasia, barrier function impairment, and cell renewal and change in metabolism, which requires folic acid for intestinal epithelial repair (Yang et al., 2016a, 2016b; Montagne et al. 2017). However, in a post-weaning diet lacking antibiotics and zinc oxide, the effect of folic acid on the growth performance and underlying mechanism on the intestinal morphology and functions in weaned piglets are still unclear. In the present study, we hypothesized that the phenotype of weaning-stress associated pathologies in diets without antibiotics and pharmacological zinc oxide is to some extent folic acid deficiency, which can be alleviated by dietary supplementation with folic acid levels above the current NRC recommendation. For piglets without antibiotics and zinc oxide in feed, we added pharmacological levels (10, 30, 60 times) of folic acid to ensure the dietary supplementation with a relative high level of folic acid, which is particularly important for ensuring the normal growth and integrity of intestine in piglets.

Therefore, the present study was conducted to evaluate the effect of dietary folic acid levels on the growth performance, intestinal morphology and functions, and intestinal epithelial cell renewal in post-weaning piglets fed an antibiotic-free and zinc oxide-free diet.

## 2 Materials and methods

The experiment design and procedures were reviewed and approved (approval number 2017-099) by the Animal Care and Use Committee of the Hunan Normal University, Changsha city, Hunan, China.

### 2.1 Animals and treatments

Twenty-eight weaned piglets (Duroc × Landrace × Yorkshire; barrow; initial average body weight of 6.73 ± 0.62 kg) at 21 days of age were selected from 10 litters based on body weight (BW) and randomly assigned to 4 treatments, with 7 pens and 1 piglet per pen and the ambient temperature was maintained at 29 ± 1 °C in the same room with the same feeding management. This study followed a fully randomized block design, where the block was based on average body weight. Twenty-eight boar piglets were selected from 10 litters with weight from 6 to 8 kg. Piglets were weighed, labelled from 1 to 28, then Excel was used to sort the weights and allot the piglets into 4 groups: A, B, C, D. Following this, adjustments were made to the average weight and standard deviation of each group and to ensure that the positions of each group in the room were scattered. The piglets were fed the same antibiotic-free and zinc oxide-free basal diets supplemented with folic acid at 0, 3, 9, and 18 mg/kg, respectively. The feed formulation (Table 1) met the nutrient specifications for piglets from 7 to 11 kg BW, the analyzed and calculated values of diets were shown in Table 2. The folic acid used in this study is a spray-dried micro-granular commercial product (Beijing, Blooming Biotechnology Co., Ltd.) containing 80% folic acid. The product uses dextrin as the carrier, which has good fluidity and is easy to mix and dilute at different ratios with the vitamin premix. A vitamin premix was prepared using a step-by-step dilution process. Then, the vitamin premixes with different folic acid contents were used for producing compound feeds. The piglets were given a high-quality diet ad libitum and free access to water throughout the study. Initial body and final body weight were calculated to evaluate growth performance.

**Table 1:** Ingredients of piglet diets (%, as fed basis).

Ingredients	Content
Corn	39.7
Extruded corn	20.0
Soybean meal, 44% CP	9.0
Fish meal, 63% CP	7.0
Spray dried porcine plasma	5.0
Whey powder	9.0
Glucose	3.0
Soybean oil	3.8
Limestone	1.05
Choline chloride	0.1
Antioxidant premix^1^	0.05
Citric acid	0.5
NaCl	0.1
Mineral premix^2^	0.15
l-Lys HCl	0.45
dl-Met	0.20
l-Thr	0.14
l-Trp	0.02
Vitamin premix^3^	0.74
Total	100

1 Supplied 50 mg ethoxyquin per kilogram of feed.

2 Supplied the following per kilogram of feed: 150 mg Fe (FeSO_4_), 100 mg Zn (ZnSO_4_), 30 mg Mn (MnSO_4_), 25 mg Cu (CuSO_4_), 0.5 mg I (KIO_3_), 0.3 mg Co (CoSO_4_), and 0.3 mg Se (Na_2_SeO_3_).

3 Supplied the following per kilogram of feed: 2,200 IU vitamin A, 220 IU vitamin D_3_, 0.5 mg vitamin K_3_, 0.0175 mg vitamin B_12_, 3.5 mg riboflavin, 30 mg niacin, 10 mg d-pantothenic acid, 0.05 mg biotin, 0.3 mg thiamine, and 7 mg pyridoxine.

**Table 2:** Chemical composition of piglet diets (%, as fed basis).

Chemical composition	Supplement folic acid level, mg/kg			
	0	3	9	18
Analyzed values^1^				
DM	89.62 ± 0.05	90.22 ± 0.20	89.33 ± 0.13	89.88 ± 0.15
CP	18.50 ± 1.24	18.25 ± 1.28	18.36 ± 1.10	18.20 ± 0.91
Calcium	0.80 ± 0.02	0.78 ± 0.03	0.81 ± 0.02	0.82 ± 0.03
Phosphorus	0.38 ± 0.02	0.37 ± 0.02	0.36 ± 0.02	0.36 ± 0.02
Folic acid, mg/kg	0.20 ± 0.02	3.21 ± 0.03	9.23 ± 0.02	18.20 ± 0.03
Calculated values^2^				
ME, kcal/kg	3,393	3,393	3,393	3,393
Lys	1.37	1.37	1.37	1.37
Met + Cys	0.75	0.75	0.75	0.75
Thr	0.81	0.81	0.81	0.81
Trp	0.22	0.22	0.22	0.22

1 The dry matter (DM), crude protein (CP), calcium, phosphorus and folic acid were analyzed values, means ± SD.

2 The concentrations of metabolizable energy, and amino acids were calculated according to the composition of feed ingredients used in swine in NRC 2012. Vitamin and trace element concentrations met the requirements according to NRC 2012.

### 2.2 Sample treatment and collection

At the end of the 14-day treatment, a 10-mL blood sample was harvested from the anterior vena cava and serum samples separated after centrifugation at 3,000 × *g* for 10 min under 4 °C. Serum amino acids were determined by the high-speed Amino Acid Analyzer L-8900 (Hitachi Co., Tokyo, Japan). Blood biochemical analysis was conducted by commercial kits following the manufacturer's instructions (Jiancheng Bioengineering Institute, Nanjing, China) and identified using the TBA-120FR Automatic Biochemistry Radiometer (Hitachi Co., Tokyo, Japan). Intestinal mucosa was collected from the middle part of the small intestine by gently scraping the mucosa with glass slides, then stored at −80 °C before measurement.

For feed folate analysis, folic acid in feeds was determined using high performance liquid chromatography (Agricultural industry standards of the People's Republic of China NY/T 2895-2016). Serum folate analysis was conducted by Roche kits following the manufacturer's instructions and identified using the Roche cobas 601 electrochemical immunoassay analyzers (Roche Ltd., Basel, Switzerland).

### 2.3 Fecal score

Fecal consistency was monitored, and the scores were recorded every day during this experiment and quantified using a scale ranging from 0 to 3 with 0 = hard feces, 1 = normally shaped feces, 2 = shapeless (loose) with liquid (soft) feces and 3 = severe diarrhea with watery feces (Liu et al., 2008).

### 2.4 Intestinal histomorphology analysis

The intestines were removed and approximately 2 cm of the jejunum was collected from the middle part of the small intestine, then placed in a 4% neutral buffer formalin solution. Specimens of the cross-sections of the intestinal segments identified above were embedded in low-melt paraffin wax and cut into 4-μm thick histological sections for hematoxylin and eosin (H&E) staining. The tissue sections were measured under a microscope using a 40× combined magnification and an image processing and analysis system (Leica Imaging Systems Ltd., Cambridge, UK). The Program Image-pro Plus 6.0 (Media Cybernetics, Inc., Georgia, USA) was used to determine the villus height (VH), crypt depth (CD) and villus height-to-crypt depth (VH:CD) ratio. At least 25 villous samples with intact lamina propria were blindly selected and examined for measurement.

### 2.5 Assay for intestinal disaccharidase activities

The disaccharidase activity of mucosal scrapings was assessed using a modified glucose oxidase peroxidase enzyme system. Jejunal mucosal tissue samples were homogenized in saline and centrifuged (2,500 × *g*, 4 °C, 10 min) to produce the supernatant. The enzyme sucrase, lactase and maltase activities were analyzed using commercial kits (Jiancheng Bioengineering Institute, Nanjing, China) and Synergy HTX Multi-Mode Microplate Reader (BioTek, Vermont, USA) following the manufacturer's instructions. The concentration of protein was also analyzed also using commercial bicinchoninic acid assay kits (Jiancheng Bioengineering Institute, Nanjing, China) and Synergy HTX Multi-Mode Microplate Reader (BioTek, Vermont, USA) according to the manufacturer's instructions. Throughout this paper, disaccharidase activity has been expressed as units per milligram protein of the mucosal sample.

### 2.6 Quantitative real-time PCR (RT-PCR) analysis

RNA isolation and RT-PCR analysis of jejunal mucosa samples was performed according to the previous study (Wang et al., 2019). Briefly, total RNA was isolated from liquid nitrogen frozen jejunal mucosa samples using RNAiso Plus (Takara, Dalian, China). The RNA quality was then checked using 1% agarose gel electrophoresis, and the concentration and purity were measured using a Synergy HTX Multi-Mode Reader (BioTek, Vermont, USA). After removing trace DNA by incubation with DNase (Takara, Dalian, China), 1 μg of RNA was reverse transcribed to cDNA using PrimeScript RT Master Mix (Perfect Real Time; Takara, Dalian, China) according to the manufacturer's instructions. RT-PCR was performed with a final volume of 10 μL (containing 5 ng of cDNA, 5 μL SYBR Green mix, 0.2 μL ROX Reference Dye [50×], and 0.2 μL each of both forward and reverse primers) at 95 °C for 10 s, 40 cycles of denaturation (95 °C for 5 s, and 60 °C for 20 s) using a QuantStudio 5 Real-Time PCR System (Thermo Fisher Scientific Inc., Rockford, IL, USA). This was followed by a melting curve program (from 60 to 99 °C with a heating rate of 0.1 °C) where fluorescence was collected, and all reactions were run in triplicate. The housekeeping gene β-actin was used as an internal control in each sample to normalize the expression of the target gene (Table 3). Due to the wide distribution of β-actin in various tissues, the amino acid sequence is highly conservative, the expression level varies little among different tissues, and the expression level is high. Relative expression of target genes was calculated according to the formula 2^−ΔΔCt^, where ΔΔCt = (Ct _Target_ – Ct _β-actin_) _treatment_ – (Ct _Target_ – Ct _β-actin_) _control_. The ideal internal reference has the following conditions: 1) Large expression; 2) Stable expression in different types of cells and tissues; 3) The expression level has nothing to do with the cell cycle and cell activation; 4) Not subject to any endogenous or the influence of exogenous factors; 5) The absence of pseudogenes to avoid the contamination and amplification of genomic DNA. Housekeeping gene beta-actin was selected and applied in our study. Previous studies have reported that β-actin must be used in isolation as an endogenous control (Darwish et al., 2010; Radonić et al., 2004). Beta-actin is widely distributed in various tissues and cells of mammalian animals, where the amino acid sequence is highly conservative (Nicot et al., 2005; Cornejo et al., 2010). The many intestine-related experiments we have previously conducted revealed that results are reliable when β-actin was used alone (Zou et al., 2019). Additionally, pretests were done that showed β-actin alone can be used. Therefore, the housekeeping gene β-actin was selected and employed in our study.

**Table 3:** Primers used for quantitative reverse transcription PCR.

Gene	Primers	Sequence (5' to 3')	Size, bp	GenBank accession No.
β-Actin	Forward	AGTTGAAGGTGGTCTCGTGG	216	XM_021086047.1
	Reverse	TGCGGGACATCAAGGAGAAG		
*Slc1a1*	Forward	GCTGTGCTGAAGAGAAGAA	181	NM_001164649.1
	Reverse	GTGGCGGTGATACTGATAG		
*Slc6a19*	Forward	CACAACAACTGCGAGAAG	152	XM_003359855.4
	Reverse	TTGATAAGCGTCAGGATGT		
*Slc2a2*	Forward	AAGTCGAGGCCTATGATCTGACTAA	161	NM_001097417.1
	Reverse	GGAAGAGGCATATCAGGACTCTACT		
*Slc7a1*	Forward	CCCCTGTGGTAGCGATGCAGTCA	240	XM_021065162.1
	Reverse	CTGGGCTTCATAATGGTGTCAGGAT		
*TNF-α*	Forward	ACAGGCCAGCTCCCTCTTAT	102	NM_214022.1
	Reverse	CCTCGCCCTCCTGAATAAAT		
*INF-γ*	Forward	CCATTCAAAGGAGCATGGAT	146	NM_213948.1
	Reverse	GAGTTCACTGATGGCTTTGC		
*IL-1β*	Forward	CCTGGACCTTGGTTCTCT	123	XM_021085847.1
	Reverse	GGATTCTTCATCGGCTTCT		

### 2.7 Immunohistochemistry for Ki67 and shedding rate

Four-μm thick histological section of each jejunum specimen were obtained as described above and placed on polyline-coated glass slides. The slides were dewaxed in xylene twice for 10 min and rehydrated in a descending ethanol series, from 100% descending to 95% and 85%, and finally to 70% ethanol with 5 min intervals. The sections were washed 3 times in PBS buffer (5 min each) and then heated twice (5 min each) in an aqueous sodium citrate solution (0.01 mol/L, pH 6.0) to retrieve antigen epitopes, and then washed another 3 times (10 min each) in PBS buffer. Endogenous peroxidase was inhibited with 3% hydrogen peroxide in methanol for 10 min and then the sections were washed for 15 min in PBS buffer. During incubation for 30 min at 37 °C, 5% bovine serum albumin (BSA; Boster Biological Technology Co. Ltd., Wuhan, China) was used at 1:10 dilution to block non-specific bindings. After incubation with the Ki-67 antibody (ab15580, Abcam, Cambridge, UK; 1:600 dilution) overnight, the sections were thrice washed in the PBS buffer and treated with a goat anti-rabbit IgG secondary antibody (ZSGB-BIO, Beijing, China) for 45 min at 37 °C. Three 5-min washes were performed in PBS buffer after each step save the blocking step. Positive cells were visualized using a diaminobenzidine (DAB) kit (ZSGB-BIO, Beijing, China) according to the manufacturer's instructions. Fifteen microscope fields of each sample were captured using an optical microscope at magnification 20× (Leica DM3000, Leica Microsystems, Wetzlar, Germany). The number of Ki-67 positive cells in each field was calculated using the Image-Pro Plus 6.0 software (Media Cybernetics, Inc. Georgia, USA; Wang et al., 2018; Chen et al., 2019). The number of villi with shedding cells were counted using the above H&E-stained section and at least 100 villi of each sample were blindly selected. The shedding rate was termed as the number of villi with shedding cells per 100 villi according to the method of a previous study (Bullen et al., 2006).

### 2.8 Statistical analysis

All results except fecal scores were performed using SPSS software (version 22.0; IBM Corp., Chicago, IL, USA). Statistical analysis among the groups was performed using one-way ANOVA analysis followed by Duncan's multiple-range test for comparing the differences among treatments. Linear and quadratic contrasts were used to examine the effects of dietary treatment (dietary supplement with 0, 3, 9, 18 mg/kg of folic acid in the basal diet). The results are presented as the mean and the standard error of the mean (SEM) in the tables (*n* = 7). Diarrhea rate was analyzed using the Fishers Exact Test procedure of SAS (version 6.12; SAS Inst. Inc., Cary, NC). *P*-value < 0.05 was considered statistically significant and 0.05 < *P* < 0.1 was considered as tending towards significance.

Two-linear broken-line regression model was estimated based on group means relative to dietary folic acid concentration (*n* = 7). It can be written as *y* = *L* + *U* × (*R* − *x*) for *x* < *R*, (*R* − *x*) is defined as zero when *x* > *R*, where the optimal demand is the breakpoint, and *L* represents the *Y*-intercept of the breakpoint, minus *U* represents the slope, *R* represents the *X*-intercept of the breakpoint (Robbins et al., 2006). Parameter estimates are presented as means ± SE to indicate the precision of estimation, and breakpoint (*X*-intercept [folic acid mg/kg diet] ± SE, *Y*-intercept ± SE) of the two-linear broken-line regression model as determined by Proc NLIN (SAS 9.3). *P* ≤ 0.05 was considered statistically significant (Brugger et al., 2014, 2019).

Using the following code (Robbins et al., 2006):

Data one;
Input x y;
Datalines;
Treatment Mean-value;
Data fill; ∗Generates multiple x values to facilitate graph of predicted values;
Do x = 0.22 to 18.22 by 0.22; y = .; output; end;
Run;
Data one; set one fill; run;
Proc sort data = one; by x;
Proc nlin data = one; ∗straight broken-line;
Parameters L = 0.14 U = −1600 to 1600 by 1 R = 4;
z1 = (x < R) ∗ (R - x);
model *y* = L + U ∗ (z1);
output out = ppp p = predy;
run;
proc gplot;
title2 'Two linear broken lines';
goptions hpos = 35 vpos = 35 ftext = swiss;
symbol1 v = dot c = black;
symbol2 i = join v = none c = black;
plot y∗x predy∗x/overlay;
run;

Notes for this code: “Treatment mean-value” were according to the different nutrient levels and the mean of indexes. Parameters L, U, and R were predicted according to the mean of the indexes of trial.

The effect size was calculated as 0.8 for the present experiment based on plasma folate status parameters (Yu et al., 2010). Based on these assumptions, with α = 0.05, a power of 0.90, it has been calculated that total sample size was 28. The G∗Power 3.1.9.2 was used to run the power analysis.

## 3 Results

### 3.1 Growth performance and fecal score in piglets

The effects of folic acid on the growth performance and fecal score are presented in Table 4. The initial body weight (IBW) of piglets showed no difference. The ADG (*P* = 0.018) and ADFI (*P* = 0.032) of weaned piglets expressed a significant linear contrast. However, no significant difference was found in the final body weight and G: F among the 4 treatments.

**Table 4:** Effects of different folic acid levels on growth performance in post-weaning piglets.

Item	Supplement folic acid level, mg/kg				SEM	*P*-value	Contrast, *P*-value	
	0	3	9	18			Linear	Quadratic
Initial BW, kg	6.73	6.71	6.72	6.74	0.12	1.000	0.980	0.954
Final BW, kg	7.49	7.67	8.11	8.02	0.16	0.500	0.173	0.692
ADG, g	55	68	99	92	7	0.069	0.018	0.424
ADFI, g	207	221	261	254	10	0.121	0.032	0.555
G:F, g/g	0.293	0.338	0.377	0.357	0.017	0.371	0.588	0.732
Diarrhea rate, %	25.71^a^	23.81^a^	14.29^b^	22.86^a^		0.191		

BW = body weight; ADG = average daily gain; ADFI = average daily feed intake; G:F = gain-to-feed ratio.

^a, b^ Means in the same row followed by different superscript letters indicate significant differences (*P* < 0.05).

### 3.2 Serum biochemical parameters and free amino acids

Serum alanine aminotransferase activity (*P* = 0.015), as well as urea nitrogen (BUN) content (*P* = 0.001; linear, *P* < 0.001) and the serum folate (SF) concentrations (*P* = 0.001; linear, *P* < 0.001) expressed a significant linear contrast. However, there were no significant differences in the serum total protein, albumin, creatinine, glucose, cholesterol contents, aspartate transaminase, and hepatic lipase (Table 5). Dietary supplementation with folic acid increased the serum free Asp (*P* = 0.034; linear, *P* = 0.021) and Hyp (*P* = 0.010; linear, *P* = 0.030; quadratic, *P* = 0.011) concentrations, and decreased the serum free Glu (*P* = 0.030; linear, *P* = 0.009), Val (*P* = 0.010; linear, *P* = 0.002), Met (*P* = 0.002; linear, *P* = 0.001), Ile (*P* = 0.016; linear, *P* = 0.024), Leu (*P* = 0.009; linear, *P* = 0.001), Tyr (*P* = 0.001; linear, *P* < 0.001), and Lys (*P* = 0.049; linear, *P* = 0.007) concentrations (Table 6). There were no significant differences among the serum free Thr, Sar, Gly, Ala, Phe, Orn, His, and Arg concentrations among the 4 dietary treatments (Table 6).

**Table 5:** Effects of different folic acid levels on serum biochemical parameters in post-weaning piglets.

Item	Supplement folic acid level, mg/kg				SEM	*P*-value	Contrast, *P*-value	
	0	3	9	18			Linear	Quadratic
TP, g/L	51.49	49.46	49.96	51.31	0.66	0.659	0.998	0.225
ALB, g/L	32.16	31.64	32.36	31.23	0.68	0.943	0.749	0.832
ALT, U/L	24.41	25.51	28.80	31.79	1.16	0.095	0.015	0.667
AST, U/L	36.71	40.29	48.86	43.43	2.53	0.395	0.217	0.383
BUN, mmol/L	4.63^a^	3.60^b^	2.97^b^	2.83^b^	0.20	0.001	<0.001	0.159
CREA, μmol/L	71.29	60.71	61.29	62.86	2.11	0.259	0.192	0.865
GLU, mmol/L	4.94	4.43	4.79	4.16	0.17	0.358	0.192	0.865
TG, mmol/L	0.48	0.44	0.43	0.37	0.02	0.313	0.075	0.849
CHOL, mmol/L	1.94	1.99	1.98	2.07	0.07	0.938	0.570	0.878
LIPC, U/L	3.57	3.49	3.44	3.29	0.08	0.646	0.220	0.825
Folate, μg/L	24.41^b^	52.03^a^	47.92^a^	61.79^a^	3.69	0.001	<0.001	0.221

TP = total protein; ALB = albumin; ALT = alanine aminotransferase; AST = aspartate transaminase; BUN = urea nitrogen; CREA = creatinine; GLU = glucose; TG = triglyceride; CHOL = cholesterol; LIPC = hepatic lipase.

^a, b^ Means in the same row followed by different superscript letters indicate significant differences (*P* < 0.05).

**Table 6:** Effects of different folic acid levels on serum free amino acids (μg/mL) in post-weaning piglets.

Item	Supplement folic acid level, mg/kg				SEM	*P*-value	Contrast, *P*-value	
	0	3	9	18			Linear	Quadratic
Tau	42.48	44.38	44.73	43.02	1.00	0.848	0.838	0.399
Urea	178.92^a^	88.62^b^	119.23^b^	113.68^b^	9.67	0.002	0.024	0.010
Asp	4.34^a^	3.72^a^	4.49^a^	6.27^b^	0.34	0.034	0.021	0.644
Thr	16.43	19.42	16.31	17.21	1.07	0.735	0.940	0.644
Glu	42.57^a^	45.50^a^	34.19^ab^	30.40^b^	2.12	0.030	0.009	0.379
Sar	0.56	0.73	0.50	0.70	0.07	0.591	0.735	0.885
Gly	77.41	69.86	93.09	81.06	4.19	0.254	0.368	0.787
Ala	45.52	49.85	48.63	47.13	1.64	0.833	0.819	0.404
Val	25.58^a^	23.27^ab^	19.30^b^	19.56^b^	0.84	0.010	0.002	0.369
Met	9.13^ab^	10.83^a^	6.30^bc^	2.64^c^	0.88	0.002	0.001	0.064
Ile	13.59^ab^	15.73^a^	12.50^b^	11.15^b^	0.56	0.016	0.024	0.082
Leu	26.13^a^	23.06^ab^	21.02^b^	20.54^b^	0.71	0.009	0.001	0.283
Tyr	13.67^a^	13.08^a^	10.69^b^	9.23^b^	0.47	<0.001	<0.001	0.534
Phe	0.25	0.23	0.11	0.25	0.04	0.438	0.705	0.271
Orn	8.11	8.31	9.77	7.80	0.52	0.558	0.911	0.318
Lys	37.14^a^	29.24^ab^	24.31^b^	21.21^b^	2.21	0.049	0.007	0.553
His	13.20	12.53	10.66	11.10	0.60	0.412	0.150	0.650
Hyp	12.24^a^	14.60^ab^	16.90^b^	14.39^ab^	0.52	0.010	0.030	0.011
Arg	26.16	31.10	24.38	26.15	1.18	0.293	0.497	0.508

^a, b^ Means in the same row followed by different superscript letters indicate significant differences (*P* < 0.05).

### 3.3 Intestinal morphology

The VH, CD, VH:CD ratio, and villus surface area (VSA) in the jejunum of piglets are shown in Table 7. Dietary supplementation with folic acid increased the VH (*P* = 0.003; linear, *P* = 0.001), VH:CD ratio (*P* = 0.002; linear, *P* = 0.001), and VSA (*P* = 0.026; linear, *P* = 0.010). However, no significant difference was observed in jejunal CD among the 4 groups.

**Table 7:** Effects of different folic acid levels on jejunal mucosal histomorphology in post-weaning piglets.

Items	Supplement folic acid level, mg/kg				SEM	*P*-value	Contrast, *P*-value	
	0	3	9	18			Linear	Quadratic
Villus height, μm	247^b^	290^a^	314^a^	311^a^	8	0.003	0.001	0.077
Crypt depth, μm	305	299	277	293	5	0.196	0.182	0.249
VH:CD ratio, μm/μm	0.81^c^	0.95^bc^	1.14^a^	1.07^ab^	0.04	0.002	0.001	0.082
Surface area, μm^2^	91,868^b^	117,210^a^	117,908^a^	120,723^a^	4,001	0.026	0.010	0.122

VH:CD ratio = the villus height-to-crypt depth ratio.

^a, b^^, c^ Means in the same row followed by different superscript letters indicate significant differences (*P* < 0.05).

### 3.4 Jejunal mucosal disaccharide enzyme activity and mRNA expression

Dietary supplementation with folic acid significantly increased the activities of lactase (*P* = 0.001; linear, *P* = 0.001) and sucrase (*P* = 0.021; linear, *P* = 0.010) in the jejunal mucosa of weaned piglets (Table 8). The mRNA expression of *SLC6a19* was a significant linear contrast (*P* = 0.023) in the dietary folic acid groups. However, the expression of *SLC7a1* (*P* = 0.021) and *TNF-α* (*P* = 0.038) was a significant linear contrast in dietary folic acid groups (Table 9). No differences were observed in the mucosal maltase activity, and the *SLC1a1*, IFN-γ, and IL-1β mRNA abundance among these groups (Table 8, Table 9).

**Table 8:** Effects of different folic acid levels on disaccharide enzyme activity of jejunal mucosal in post-weaning piglets (U/mg protein).

Items	Supplement folic acid level, mg/kg				SEM	*P*-value	Contrast, *P*-value	
	0	3	9	18			Linear	Quadratic
Lactase	6.6^a^	8.8^a^	19.5^b^	26.8^b^	2.3	0.001	0.001	0.458
Sucrase	15.1^a^	24.6 ^ab^	38.2^b^	31.5^b^	2.9	0.021	0.010	0.115
Maltase	11.9	9.5	9.9	15.3	1.6	0.571	0.465	0.238

^a, b^ Means in the same row followed by different superscript letters indicate significant differences (*P* < 0.05).

**Table 9:** Effects of dietary folic acid levels on mRNA expression in the jejunal mucosa of post-weaning piglets.

Item	Supplement folic acid level, mg/kg				SEM	*P*-value	Contrast, *P*-value	
	0	3	9	18			Linear	Quadratic
*SLC1a1*	1.31	1.06	1.65	0.89	0.16	0.406	0.665	0.450
*SLC2a2*	0.63	1.03	1.14	1.09	0.09	0.171	0.062	0.184
*SLC6a19*	0.92	0.93	1.46	1.62	0.13	0.119	0.023	0.777
*SLC7a1*	2.08	1.57	1.07	0.97	0.18	0.111	0.021	0.564
*TNF-α*	2.01	1.49	1.49	1.04	0.15	0.175	0.038	0.904
*IFN-γ*	1.11	1.23	1.11	0.66	0.10	0.221	0.111	0.173
*IL-1β*	1.18	0.96	1.09	0.66	0.10	0.284	0.118	0.589

*Slc1a1=* solute carrier family 1 member 1; *Slc2a2=* solute carrier family 2 member 2; *Slc6a19=* solute carrier family 6 member 19; *Slc7a1* = solute carrier family 7 member 1; *TNF-α* = tumor necrosis factor-α; *IFN-γ* = interferon-γ; *IL-1β* = interleukin-1 β.

### 3.5 Jejunal epithelial cells renewal

**Fig. 1:**
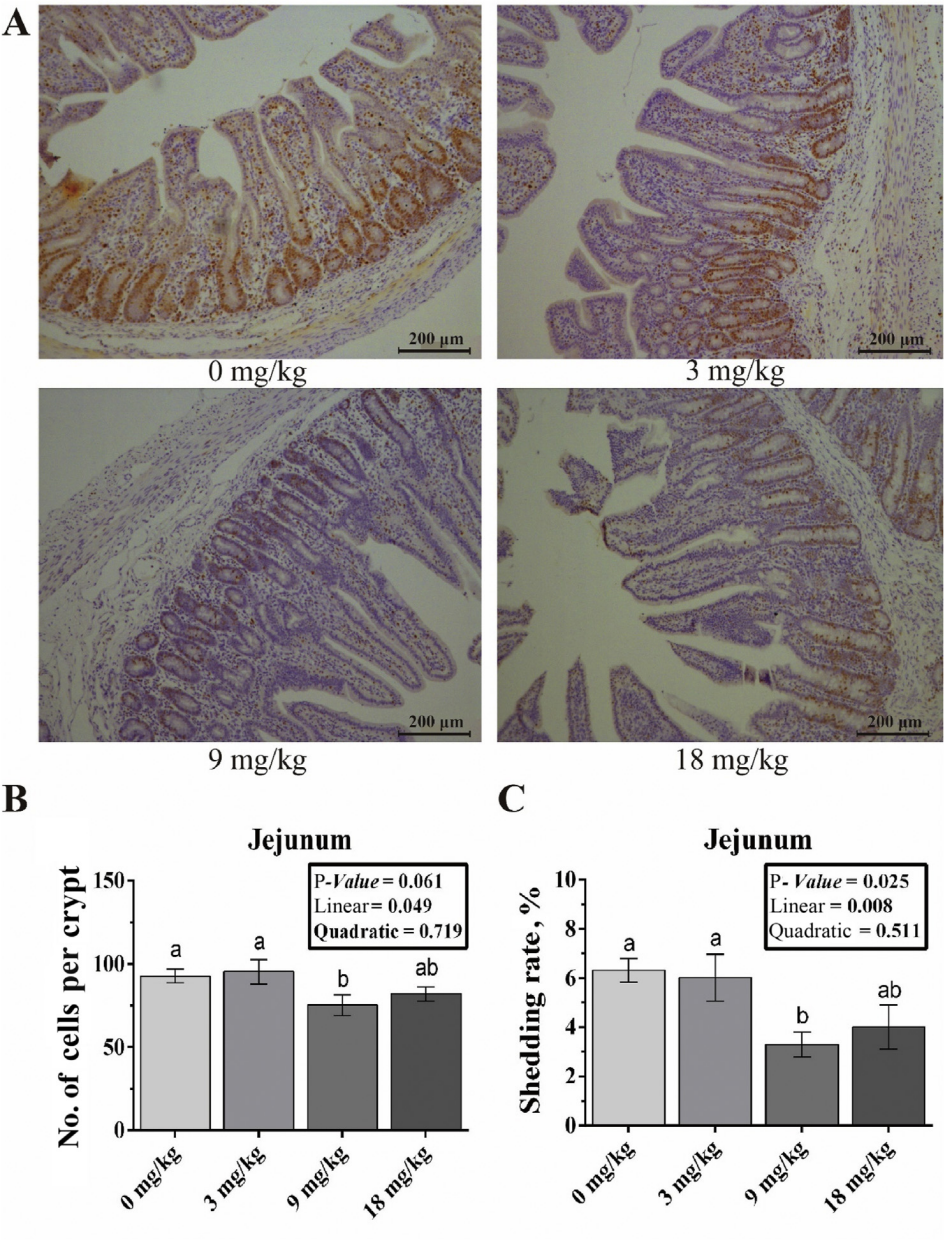
Folic acid supplementation in weaned piglet intestinal cell renewal. (A) The immunohistochemistry (IHC) staining with Ki67 antibody of the jejunum at magnification 200×. (B) The number of Ki67 positive cells of crypt within jejunum in weaned piglets in different folic acid levels. (C) The shedding rate of epithelial cells within the jejunum of weaned piglets with different folic acid levels. ^a, b^ Different letters indicate significant differences (*P* < 0.05). Data are expressed as means ± SEM (*n* = 7). Each treatment was extra supplemented with 0, 3, 9, 18 mg/kg folic acid, respectively.

The effects of dietary folic acid on the proliferation of jejunal epithelial cells and cell shedding are presented in Fig. 1. The number of Ki67 positive cells, and the shedding rate of epithelial cells had a significant linear contrast (*P* = 0.049, and 0.008, respectively) in the dietary folic acid groups.

### 3.6 Result of two linear broken line regression

**Fig. 2:**
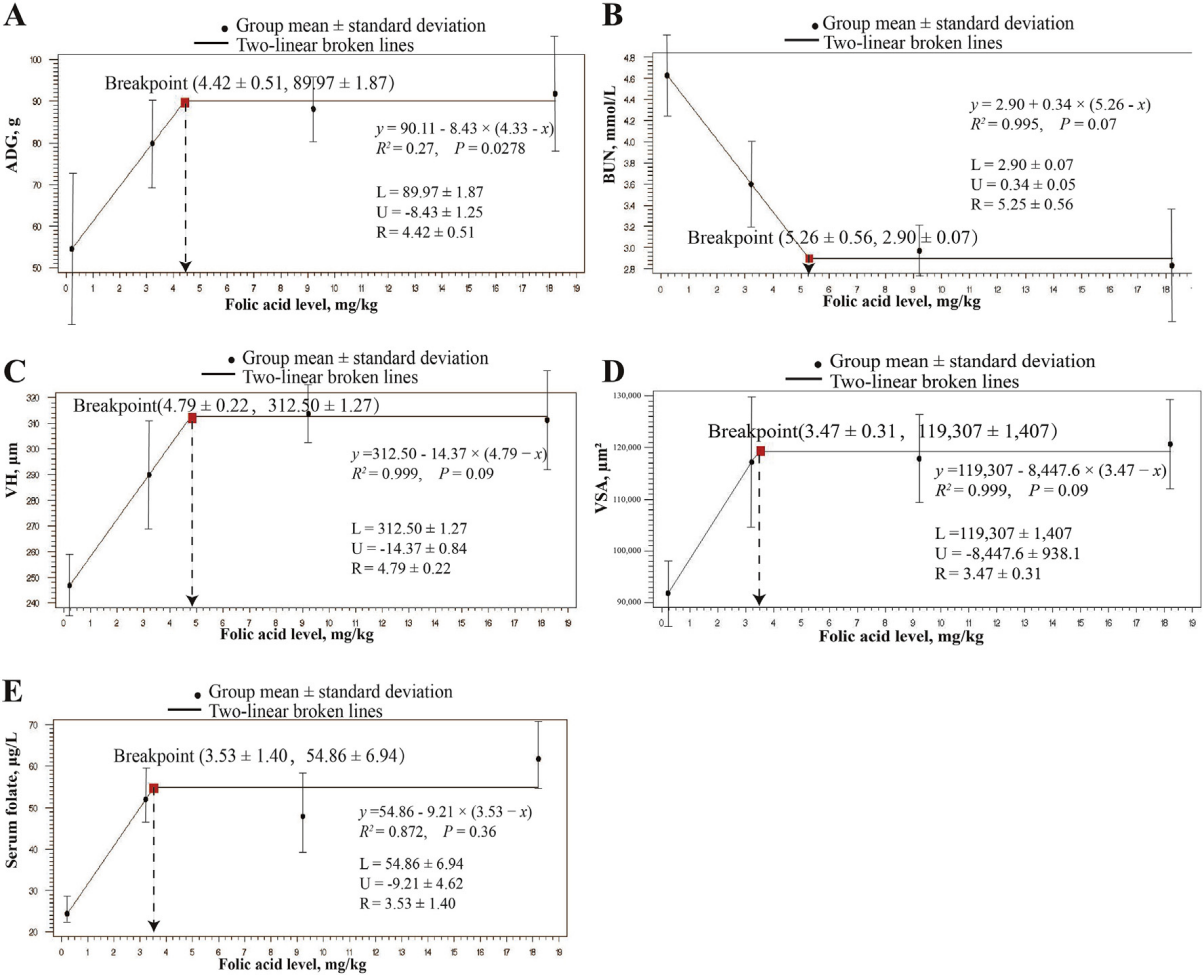
The two-linear broken-line regression model was estimated based on group means relative to dietary folic acid concentration (0.22, 3.22, 9.22, 18.22 mg/kg) in weaned piglets *(n* = 7). Response of ADG (A), BUN content (B), VH (C), VSA (D), serum folate concentration (E) in weaned piglets fed diets with different folic acid concentrations and the respective regression curves. Notes: Values of *L*, *U*, and *R* are means ± SE. *L* = the Y-intercept of the breakpoint; minus *U* = the slope; *R* = the *X*-intercept of the breakpoint; ADG = average daily gain; BUN = serum urea nitrogen; VH = villus height; VSA = villus surface area.

As shown in Fig. 2, the breakpoints were based on significant estimates for the *X* and *Y* intercepts of respective thresholds [(*X*-intercept (folic acid mg/kg diet) ± SE, *Y*-intercept ± SE); (4.42 ± 0.51, 89.97 ± 1.87 g/d), (5.26 ± 0.56, 2.90 ± 0.07 mmol/L), (4.79 ± 0.22, 312.50 ± 1.27 μm), (3.47 ± 0.31, 119,307 ± 1407 μm^2^) and (3.53 ± 1.40, 54.86 ± 6.94 μg/L) for ADG, BUN, VH, VSA and SF, respectively].

Above these breakpoints, the response of ADG, VH, VSA, and SF plateaued in response to the changes in dietary folic acid concentration whereas BUN followed a negative correlated response to the dietary folic acid concentration (*R*^2^ = 0.995, *P* = 0.07). Below the respective breakpoints, the response of ADG, VH, VSA, and SF correlated positively, and respectively, to the dietary folic acid concentration (*R*^2^ = 0.995, 0.999, 0.999, and 0.872, *P* = 0.09, 0.09, 0.09, and 0.36, respectively). Slopes of the ADG, BUN, VH, VSA, and SF response of the linear regression curves are 8.43 ± 1.25, −0.34 ± 0.05, 14.37 ± 0.84, 8447.6 ± 938.1, and 9.21 ± 4.62, respectively.

## 4 Discussion

The intestine is not only an organ for digestion and absorption, but also an important immune organ. Improving intestinal morphology and integrity is the main way to improve the growth performance of weaned piglets. In line with the intestinal morphology and functions improvement, dietary supplementation with folic acid 9 and 18 mg/kg groups significantly increased the ADG in weaned piglets. Using the broken-line model, the ADG also responded positively and significantly to changes below the dietary folic acid 4.33 mg/kg concentration. Moreover, the BUN was significantly lower in these folic acid treatment groups as compared to the control group, and the dietary folic acid concentration of a lower BUN was 5.26 mg/kg. Our results indicated that dietary supplementation with high-dose folic acid (3, 9 and 18 mg/kg) can increase the utilization of proteins, by promoting body protein synthesis and deposition. At the same time, dietary supplementation with 9 and 18 mg/kg folic acid can significantly decrease some essential amino acids content in the blood, such as Lys, Met, and branched-chain amino acids, which further supported the above conclusion. Therefore, dietary supplementation with folic acid may improve the growth performance of weaned piglets by both increasing the nutrient digestibility and deposition.

Folic acid plays a key role in the methylation cycle and DNA biosynthesis cycle (Scaglione and Panzavolta, 2014). The deficiency of folic acid in diet is likely to impair cell division and reduce the capacity of protein synthesis. These effects are most pronounced in rapidly growing tissues such as red blood cells, intestinal mucosa, embryos, and fetuses (Harper et al., 1996). Regarding poultry, some studies have shown that a higher concentration of folic acid was required in a higher protein diet or when sucrose is the sole source of carbohydrates. Our results showed that serum Met concentration of folic acid treatment groups (9 and 18 mg/kg) significantly declined more than other amino acids compared to the control group. This may be because folic acid plays an important role in regulating Met metabolism and high folic acid promotes Met catabolism (Halsted et al., 2002). The serum alanine aminotransferase activity that is significantly high in the 18 mg/kg folic acid group may affect liver metabolism. Serum folate concentration in the control group was significantly lower than folic acid treatment groups (3, 9, and 18 mg/kg), and in the broken-line model, the SF responded positively to changes below the dietary folic acid 3.53 mg/kg concentration. There were no significant differences in other serum biochemical parameters, such as total protein, glucose, and cholesterol, which indicated that additional supplementation with folic acid may not affect other nutrients or organs metabolism. Additionally, dietary supplementation with 18 mg/kg folic acid down-regulated in the mRNA expression of *TNF-α*, which suggests that folic acid can improve the intestinal integrity of weaned piglets by reducing the inflammatory response.

During the process of weaning, the piglets were subjected to a lot of stressors (e.g., change in nutrition and new environment), leading to post-weaning ‘growth check’, mainly because feed intake had decreased remarkably, and there was an acute deficiency of nutrients (Pluske et al., 1997). Weaning led to intestinal villus atrophy and crypts hyperplasia, and a lower VH:CD ratio within 3 to 5 days, resulting in a decrease in the digestive and absorptive capacity which contributed to post-weaning diarrhea (Hampson, 1986; Miller et al., 1986). The previous study showed that the addition of folic acid can revert the changes in the structural and functional abnormalities of the small intestine in infants, caused by nutritional folic acid deficiency (Davidson and Townley, 1977). The present study revealed that folic acid treatment (3, 9, and 18 mg/kg) can significantly increase jejunal VH and VSA when compared to the control group, and using the broken-line model, VH, and VSA positively responded to changes below the dietary folic acid 4.79, and 3.47 mg/kg concentration, respectively. However, this finding was inconsistent with previous studies, in which Liu et al. (2011b) reported that although maternal folic acid supplementation modulated apoptosis-related gene expressions in the jejunum of newborn piglets, there was no significant change in the intestinal morphology. The reason for the deviation in our results may be because the weaned piglets had a low feed intake and a relative lack of folic acid, while the suckling piglets had enough folic acid from milk to maintain the intestinal morphology. Taken together, our results indicated that folic acid supplementation could improve the intestinal morphology in weaned piglets.

Intestinal morphology is correlated with gut capacity to digest and absorb the nutrients (Wang et al., 2018). Weaning stress not only affects the intestinal morphology but also impairs the intestinal digestive and absorptive functions (Montagne et al., 2007). Small intestinal disaccharidases are key enzymes in the digestion and absorption of carbohydrates in piglets, while the disaccharidases activity and the digestive capacity decrease after weaning, resulting in the occurrence of diarrhea (Miller et al., 1986; Kelly et al., 1991a; Kelly et al., 1991b, c). Consistent with the improvement in intestinal morphology, dietary supplementation with 9 and 18 mg/kg folic acid can significantly increase lactase and sucrase activity in the jejunal mucosa of weaned piglets. Our results are consistent with previous studies where the increase of folic acid supplementation enhanced the activities of intestinal enzymes and reversed the ethanol-induced inhibition of intestinal enzyme activities (Greene et al., 1974; Sesay et al., 2016). In addition, dietary supplementation with 18 mg/kg can up-regulate the mRNA expression of *SLC6a19,* a neutral amino acid transporter. Our results also indicate that additional supplementation with folic acid could improve intestinal digestive and absorptive functions. However, dietary supplementation with folic acid downregulated the mRNA expression of *SLC7a1*, a cationic amino acid transporter, in the jejunal mucosa of weaned piglets. Therefore, clarification will be needed on the mechanism of the action of folic acid on jejunal amino acid transporters expression and the effects of folic acid on nutrient digestibility. As a proinflammatory cytokine, *TNF-α* high expression has negative effects on intestinal integrity (Pié et al., 2004). Our results showed that dietary supplementation with 18 mg/kg folic acid can significantly downregulate the expression of *TNF-α,* suggesting that additional supplementation with folic acid may also decrease the signs of intestinal inflammation of weaned piglets. However, the expression of another two proinflammatory cytokines, *IFN-γ* and *IL-1β*, were not significantly affected by dietary folic acids, which may be because these proinflammatory cytokines have different expression patterns in the intestine of piglets during the post-weaning period (Pié et al., 2004).

It is well known that the normal morphology of the intestine is determined by the balance between cell proliferation and cell shedding (Miguel et al., 2017). However, weaning stress can promote the apoptosis of intestinal epithelial cells and proliferation of intestinal stem cells in the crypts (Zhu et al., 2014; Yang et al., 2016a), thereby accelerating villus atrophy and intestinal epithelial cell renewal, which results in a dysfunction of the small intestine (Pluske et al., 1997; Boudry et al., 2004; Yang et al., 2013). To evaluate the effects of folic acid on the proliferation of intestinal epithelial cells, we measured the number of Ki-67 positive cells, a reliable marker of cell proliferation (Scholzen and Gerdes, 2000; Wang et al., 2018), in the jejunum of weaned piglets. Our results showed that dietary supplementation with 9 mg/kg folic acid can significantly decrease the number of Ki-67 positive cells and the shedding rate of intestinal epithelial cells, which indicates that high doses of folic acid can alleviate the cell shedding induced by weaning stress and thus maintain the function of the intestinal epithelial cells. Therefore, additional supplementation with folic acid may maintain the balance of intestinal epithelial cell renewal to keep the intestinal morphology and function of weaned piglets intact.

## 5 Conclusion

The present study indicates that piglets supplemented with folic acid had positive growth performance and intestinal morphology during the post-weaning period. In addition, dietary supplementation with folic acid can decrease the number of Ki-67 positive cells and the shedding rate of intestinal epithelial cells. Further studies need to be conducted to find the optimal concentration of folic acid for the weaned piglets through the final estimation of the requirement threshold. In general, the present study will provide valuable guidance for adding a suitable concentration of folic acid in weaning piglets.

## Author contributions

Huansheng Yang: conceptualization, methodology, software, writing–review and editing. Lei Wang: investigation, data curation, writing-original draft preparation. Xian Tan, Huiru Wang: investigation. Qiye Wang: visualization. Pengfei Huang: resources. Yali Li: formal analysis. Jianzhong Li: project administration. Jing Huang: software, validation. Yulong Yin: supervision, funding acquisition, writing–review and editing.

## Conflict of interest

We declare that we have no financial or personal relationships with other people or organizations that might inappropriately influence our work, and there is no professional or other personal interest of any nature or kind in any product, service and/or company that could be construed as influencing the content of this paper.
